# Vaginal *Lactobacillus* Inhibits HIV-1 Replication in Human Tissues *Ex Vivo*

**DOI:** 10.3389/fmicb.2017.00906

**Published:** 2017-05-19

**Authors:** Rogers A. Ñahui Palomino, Sonia Zicari, Christophe Vanpouille, Beatrice Vitali, Leonid Margolis

**Affiliations:** ^1^Section of Intercellular Interaction, Eunice Kennedy Shriver National Institute of Child Health and Human Development, National Institutes of Health, BethesdaMD, United States; ^2^Department of Pharmacy and Biotechnology, University of BolognaBologna, Italy

**Keywords:** *Lactobacillus*, HIV-1, human tissue, lactic acid, pH

## Abstract

*Lactobacillus* species, which dominate vaginal microbiota of healthy reproductive-age women, lower the risks of sexually transmitted infections, including the risk of human immunodeficiency virus (HIV) acquisition. The exact mechanisms of this protection remain to be understood. Here, we investigated these mechanisms in the context of human cervico-vaginal and lymphoid tissues *ex vivo*. We found that all six *Lactobacillus* strains tested in these systems significantly suppressed HIV type-1 (HIV-1) infection. We identified at least three factors that mediated this suppression: (i) Acidification of the medium. The pH of the undiluted medium conditioned by lactobacilli was between 3.8 and 4.6. Acidification of the culture medium with hydrochloric acid (HCl) to this pH in control experiments was sufficient to abrogate HIV-1 replication. However, the pH of the *Lactobacillus*-conditioned medium (CM) diluted fivefold, which reached ∼6.9, was also suppressive for HIV-1 infection, while in control experiments HIV-1 infection was not abrogated when the pH of the medium was brought to 6.9 through the use of HCl. This suggested the existence of other factors responsible for HIV-1 inhibition by lactobacilli. (ii) Lactic acid. There was a correlation between the concentration of lactic acid in the *Lactobacillus*-CM and its ability to suppress HIV-1 infection in human tissues *ex vivo*. Addition of lactic acid isomers D and L to tissue culture medium at the concentration that corresponded to their amount released by lactobacilli resulted in HIV-1 inhibition. Isomer L was produced in higher quantities than isomer D and was mostly responsible for HIV-1 inhibition. These results indicate that lactic acid, in particular its L-isomer, inhibits HIV-1 independently of lowering of the pH. (iii) Virucidal effect. Incubation of HIV-1 in *Lactobacillus*-CM significantly suppressed viral infectivity for human tissues *ex vivo*. Finally, lactobacilli adsorb HIV-1, serving as a sink decreasing the number of free virions. In summary, we found that lactobacilli inhibit HIV-1 replication in human tissue *ex vivo* by multiple mechanisms. Further studies are needed to evaluate the potential of altering the spectra of vaginal microbiota as an effective strategy to enhance vaginal health. Human tissues *ex vivo* may serve as a test system for these strategies.

## Introduction

The vaginal microbiota of healthy reproductive-age women is generally dominated by *Lactobacillus* species (Ravel et al., 2011). Lactobacilli are considered to be health-promoting microorganisms since they are involved in maintaining vaginal homeostasis by preventing overgrowth of pathogenic and opportunistic organisms (Ronnqvist et al., 2006; O’Hanlon et al., 2013). Indeed, lactobacilli play a key role in the prevention of numerous urogenital diseases such as bacterial vaginosis and yeast infections as well as sexually transmitted infections, both bacterial (*Chlamydia trachomatis*, *Neisseria gonorrhoeae*, *Trichomonas vaginalis*) and viral. In particular, lactobacilli have been reported to protect against vaginal transmission of human immunodeficiency virus (HIV) (Sewankambo et al., 1997; Atashili et al., 2008; Gosmann et al., 2017).

Although many hypotheses have been formulated regarding the protective effects of lactobacilli, the exact mechanisms of HIV inhibition by vaginal lactobacilli remain to be fully elucidated. These mechanisms seem to involve production of antiviral compounds such as lactic acid, hydrogen peroxide, bacteriocins, and lectins (Aldunate et al., 2013; Petrova et al., 2013). Also, the inhibition of HIV transmission has been reported to be mediated by lactobacilli that affect vaginal epithelia, modulate bacterial vaginosis, or change local or systemic immune responses (Kaewsrichan et al., 2006; Reid et al., 2011).

Here, we investigated the role of vaginal lactobacilli on HIV type-1 (HIV-1) infection of human lymphoid tissues and of human cervico-vaginal tissues *ex vivo.* The latter *in vivo* are the first gateway for HIV-1 infection during vaginal virus transmission (Redondo-Lopez et al., 1990). These *ex vivo* systems have many advantages over the conventional single-cell cultures, as they retain the majority of cell types in the context of native tissue cytoarchitecture. Also, human tissues *ex vivo* express key cell surface molecules relevant to HIV infection, and this system does not require exogenous activation or stimulation to support productive HIV infection (Grivel and Margolis, 2009).

Here, we found that HIV-1 replication in human tissues *ex vivo* was significantly suppressed by lactobacilli, and we identified multiple mechanisms of this phenomenon.

## Materials and Methods

### *Lactobacillus* Culture Conditions

Fifteen *Lactobacillus* strains (*L. crispatus* BC1, BC3–BC8; *L. gasseri* BC9–BC14; and *L. vaginalis* BC16, BC17), isolated from vaginal swabs of healthy premenopausal women (Parolin et al., 2015), were cultured overnight at 37°C in anaerobic jars containing Gaspak EZ (Becton Dickinson) in modified medium. This modified medium contained 75% Roswell Park Memorial Institute (RPMI) 1640 medium (Gibco BRL, Carlsbad, CA, United States), supplemented with 15% fetal bovine serum (FBS), sodium pyruvate at 1 mM, non-essential amino acids at 1 mM, and 25% de Man, Rogosa, and Sharpe (MRS) broth (Difco, Detroit, MI, United States) supplemented with 0.05% L-cysteine. The turbidity levels of overnight cultures were adjusted to an optical density conversion factor (OD_600__nm_) of 0.5, corresponding to a cell concentration of 10^8^ colony forming units (CFU)/mL. Culture media were centrifuged at 4,000 × *g* for 10 min at 4°C and then filtered through a 0.22 μm membrane filter. The resultant *Lactobacillus*-conditioned medium (CM) was then used to treat tissue explants infected by HIV-1. *Lactobacillus*-cell pellets (CP) were washed in sterile saline solution (0.9% NaCl supplemented with 0.05% L-cysteine) and resuspended in antibiotic-free modified medium.

### *Ex Vivo* Tissue Cultures and HIV-1 Infection

Human cervico-vaginal tissue explants obtained from routine hysterectomy (National Disease Research Interchange, Philadelphia, PA, United States) and tonsillar tissue (Children’s National Medical Center, Washington, DC, United States) were dissected and cultured as described in Grivel and Margolis (2009) with slight modifications. Briefly, the tonsillar and mucosa layers from ecto- and endo-cervix tissues were cut in blocks of 2 mm^3^. Eighteen cervico-vaginal tissue blocks were infected with 0.4 mL of viral stock HIV-1_BaL_ (120 ng/mL p24_gag_ obtained from the Virology Quality Assurance Laboratory at Rush University, Chicago, IL, United States) for 2.5 h at 37°C in agitation. After infection, tissue blocks were washed three times with phosphate-buffered saline (PBS) and transferred at the liquid–air interface onto Gelfoam (nine blocks per well) in a 12-well plate containing RPMI 1640 medium at 1 mL/well supplemented with 15% FBS, sodium pyruvate at 1 mM, non-essential amino acids at 1 mM, gentamicin sulfate at 50 μg/mL, amphotericin B at 2.5 μg/mL. Twenty-seven tonsillar tissue blocks (nine blocks per well in 3 mL of RPMI 1640 medium supplemented as above) were placed on collagen sponge gels, and tissue blocks were infected with 7.5 μL of viral stock, on top of each block. Cervico-vaginal and tonsillar tissue were incubated at 37°C for 12 days, with replacement of culture medium every 3 days. 3TC (lamivudine at 10 μM) was used as a positive control for HIV-1 inhibition.

### *Lactobacillus* Colonization on Tonsillar Explants and Evaluation of Tissue Cell Depletion

Tonsillar tissues were colonized with 15 vaginal *Lactobacillus* strains (27 blocks per condition) at a starting inoculum of 10^4^ CFU/mL. At day 3 after inoculation with bacteria, all tissue blocks were collected and digested with collagenase IV (5 mg/mL; Gibco BRL) for 30 min with agitation in a Thermomixer at 900 rpm at 37°C. Following digestion, tissue cells were filtered with 100 μm cell strainers (Corning) and washed with 50 mL of PBS. Cells were then suspended in 1 mL of PBS and stained with 1 μl of live/dead Fixable Viability Dye eFluor 450 (EF 450, Invitrogen) for 15 min. After incubation, cells were washed and diluted in staining buffer [PBS, 1% normal mouse serum, 1% normal goat serum, 1 mM ethylenediaminetetraacetic acid (EDTA)] and stained with anti-CD3-allophycocyanin (CD3-APC) for 20 min. After surface staining, cells were permeabilized with the Fix&Perm Cell Fixation and Cell Permeabilization Kit (Invitrogen) then stained for 20 min with anti-Bcl2-PE, a mitochondrial anti-apoptotic antigen. Data were acquired with a Novocyte flow cytometer (ACEA Biosciences, CA, United States) equipped with 405, 488, and 640 nm laser lines using NovoExpress version 1.2.4 software (ACEA Biosciences) and analyzed using the same software.

### HIV-1 Infection of Human Tissues *Ex Vivo* Treated with *Lactobacillus*-CM

Cervico-vaginal and tonsillar tissue blocks were cultured in *Lactobacillus*-CM from six *Lactobacillus* strains (*L. crispatus* BC3, BC5; *L. gasseri* BC12, BC13; and *L. vaginalis* BC16, BC17), obtained as described above. Tissue blocks were pre-incubated with *Lactobacillus*-CM undiluted and diluted 1:5 with normal medium for 2 h before HIV-1 infection. After HIV-1 infection, tissue cultures were kept in the same medium (undiluted or diluted 1:5) for the next 3 days of culture, then the medium was replaced with complete RPMI 1640 medium every 3 days, and the culture was kept until day 12.

### Virucidal Effect

We carried out virucidal experiments by pre-treating HIV-1 with *Lactobacillus*-CP or *Lactobacillus*-CM. HIV-1 viral suspensions at 400 μL were mixed with 100 μL of *Lactobacillus*-CP (stock 5 × 10^8^ CFU/mL), corresponding to a final concentration of 10^8^ CFU/mL, or 100 μl of undiluted *Lactobacillus*-CM (corresponding to a final 1:5 dilution) or with 100 μL of normal medium (experimental control condition). Cultures under these three experimental conditions were then incubated for 60 min at 37°C and centrifuged at 4,000 × *g* for 10 min at 4°C. Supernatants were used to infect cervico-vaginal tissue, as described above.

### Lactic Acid Quantification, pH Measurement of *Lactobacillus*-CM, and Evaluation of Their Effect on HIV-1 Replication

We quantified titers of lactate isomers D and L from overnight-cultured *Lactobacillus*-CM using a lactate quantification assay kit according to the manufacturer’s instructions (BioAssay Systems, EFDLC-100 and EFLLC-100). Isomers D (3 mM), L (23 mM), and D + L (3 mM + 23 mM), corresponding to the average titers found in all undiluted *Lactobacillus*-CM, were tested for HIV-1 inhibition in human cervico-vaginal and lymphoid tissues. Isomers D and L at concentrations corresponding to those found in dilution 1:5 were also tested in lymphoid tissues. pH values in all *Lactobacillus*-CM, undiluted or diluted 1:5, were measured as well. Furthermore, in order to evaluate the effect of low pH on HIV-1 replication in tissues *ex vivo*, as measured in *Lactobacillus*-CM (undiluted average around pH 4 and diluted 1:5 up to pH 6.9), we evaluated HIV-1 infectivity in *ex vivo* tissue at pH 4 and pH 6.9, buffering the medium with hydrochloric acid (HCl).

### Evaluation of HIV-1 Replication

We evaluated HIV-1 replication on tissue by measuring the levels of p24_gag_ in tissue culture medium using a dynamic immunofluorescent cytometric bead assay as described by Biancotto et al. (2009).

### Statistical Analysis

We performed all statistical analyses using ANOVA test GraphPad Prism version 7 (GraphPad Prism Software Inc., San Diego, CA, United States). Results were deemed significant for *p*-values < 0.05.

## Results

### *Lactobacillus* Colonization of Tissue Explants

**FIGURE 1:**
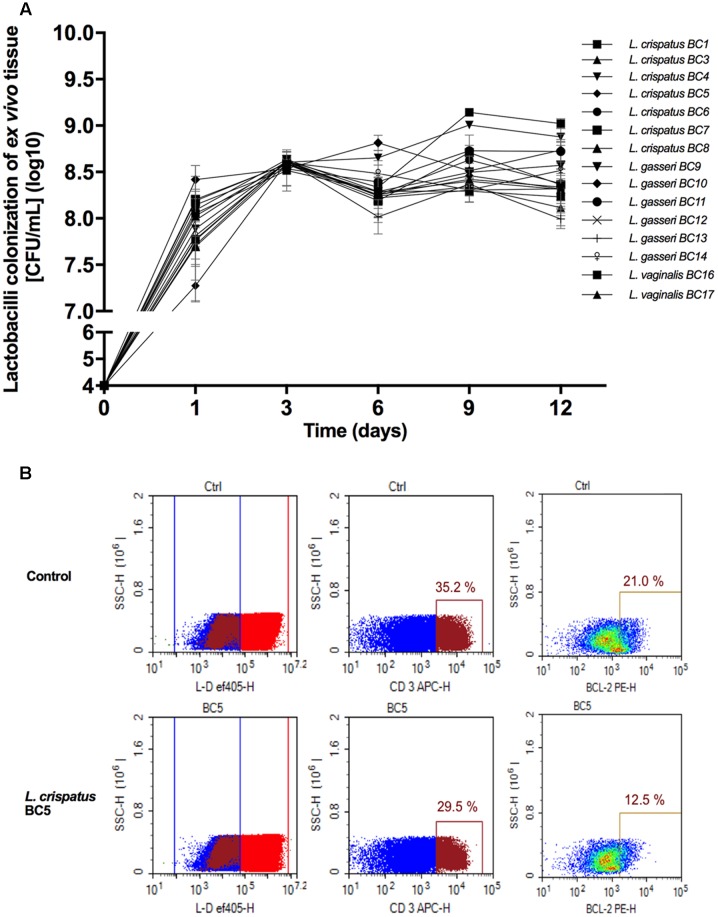
***Lactobacillus* colonization of *ex vivo* tissue. (A)** Tonsillar tissues were colonized with 15 vaginal *Lactobacillus* strains (*L. crispatus* BC1, BC3–BC8; *L. gasseri* BC9–BC14 and *L. vaginalis* BC16, BC17), at a starting inoculum of 10^4^ CFU/mL and cultured for 12 days. We evaluated *Lactobacillus* colonization every 3 days by measuring OD_600_ using a spectrophotometer. Bars represent mean ± SD from tissues of three donors. **(B)** We evaluated tissue cell depletion induced by *Lactobacillus* colonization of *ex vivo* tissues 3 days after bacterial inoculation using flow cytometry. Panels (from left to right) represent live/dead staining, CD3^+^ expression in live cells, and Bcl2 expression in CD3^+^ cells in control (upper row) and *L. crispatus* BC5-colonized tissue (lower row).

Fifteen *Lactobacillus* strains belonging to *L. vaginalis*, *L. gasseri*, and *L. crispatus* were evaluated for their capacity to colonize *ex vivo* tissue blocks. All lactobacilli colonized tissue explants with similar kinetics, reaching maximum colonization (approximately 10^8.5^ CFU/mL) after 3 days of culture, and then plateaued for the entire 12 days of culture duration (**Figure 1A**). Cell depletion in tissue explants by lactobacilli was evaluated 3 days after bacterial inoculation (**Figure 1B**). Colonization of explants with 6 out of 15 *Lactobacillus* strains, *L. crispatus* (BC3, BC5), *L. gasseri* (BC12, BC13), and *L. vaginalis* (BC16, BC17), did not result in cell depletion, as compared with control (**Figure 1B**, lower panel, BC5 representative of this group). In contrast, the colonization of tissue blocks by the remaining nine *Lactobacillus* strains, *L. crispatus* (BC1, BC4, BC6, BC7, BC8) and *L. gasseri* (BC9, BC10, BC11, BC14), resulted in a loss of T (CD3^+^) cells as well as an increase in the expression of the apoptotic marker Bcl2 (data not shown). The losses of CD3^+^ cells were not characteristics of particular species of *Lactobacillus*, as some strains of *L. crispatus*, *L. gasseri*, and *L. vaginalis* induced cell depletion while others did not. *Lactobacillus* strains that induced CD3^+^ cell depletion were not used in further experiments.

### *Lactobacillus*-CM Inhibits HIV-1 Replication

**FIGURE 2:**
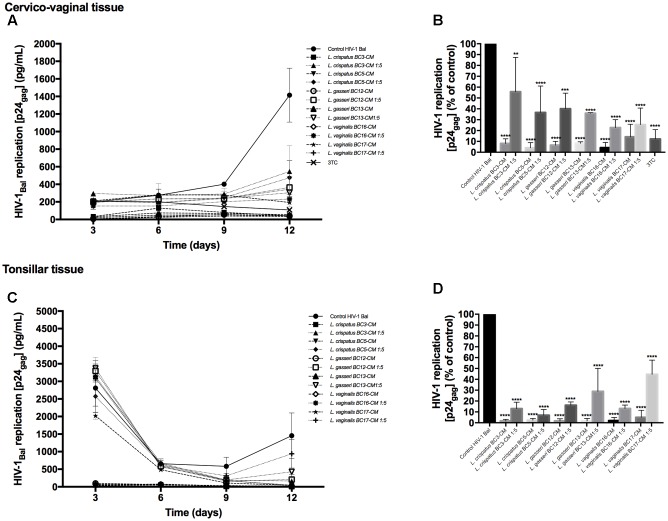
**HIV-1 infection of human tissue *ex vivo* treated with *Lactobacillus*-CM.** Cervico-vaginal **(A,B)** and tonsillar **(C,D)** tissue blocks were pre-incubated with undiluted and diluted 1:5 *Lactobacillus*-CM from six strains (*L. crispatus* BC3 and BC5; *L. gasseri* BC12 and BC13; and *L. vaginalis* BC16 and BC17). Tissue cultures were inoculated with HIV-1 and kept in the *Lactobacillus*-CM for 3 days. At day 3, the *Lactobacillus*-CM was removed and cultures were kept in regular medium until day 12 post-inoculation. **(A,C)** We evaluated the kinetics of HIV-1 replication in tissues by measuring the levels of p24_gag_ in tissue culture medium. **(B,D)** Replication of HIV-1 in *Lactobacillus*-treated tissues expressed as percentages of HIV-1 replication in untreated control (black bars). Statistical significance vs. control is presented. Bars represent mean ± SD from five tissue donors. Asterisks indicate statistical significance by one-way ANOVA multiple comparison (^∗^*p* < 0.05, ^∗∗^*p* < 0.01, ^∗∗∗^*p* < 0.001, ^∗∗∗∗^*p* < 0.0001). 3TC (dideoxythiacytidine or lamivudine) at 10 μM is a powerful HIV-1 inhibitor that we used in our study as a positive control.

To investigate the effects of metabolites secreted by lactobacilli on HIV-1 replication in *ex vivo* tissues, tissue blocks were pre-incubated with *Lactobacillus* media conditioned by *L. crispatus* (BC3, BC5), *L. gasseri* (BC12, BC13), and *L. vaginalis* (BC16, BC17), infected with HIV-1, and cultured as described in Section “Materials and Methods.” In both cervico-vaginal and lymphoid tissues undiluted *Lactobacillus*-CM suppressed replication of HIV-1 compared with the control by 91.9 ± 4.3 and 98.3 ± 1.4% (*L. crispatus* BC3, *p* < 0.0001, *n* = 5), 95.9 ± 4.8 and 97.7 ± 1.8% (*L. crispatus* BC5, *p* < 0.0001, *n* = 5), 93.5 ± 3.6 and 98.2 ± 1.9% (*L. gasseri* BC12, *p* < 0.0001, *n* = 5), 91.9 ± 1.5 and 98.3 ± 2.3% (*L. gasseri* BC13, *p* < 0.0001, *n* = 5), 95.9 ± 5.0 and 98.1 ± 2.9% (*L. vaginalis* BC16, *p* < 0.0001, *n* = 5), 85.8 ± 11.7 and 95.0 ± 6.5% (*L. vaginalis* BC17, *p* < 0.0001, *n* = 5), respectively (**Figure 2**). *Lactobacillus*-CM had an inhibitory effect on HIV-1 replication even when diluted fivefold. Depending on the *Lactobacillus* strain, inhibition of HIV-1 replication by such diluted medium was ranging from 44.3 ± 31.7% (*L. crispatus* BC3, *p* = 0.0038, *n* = 5) to 77.3 ± 7.3% (*L. vaginalis* BC16, *p* < 0.0001, *n* = 5) in cervico-vaginal tissue (**Figure 2B**) and from 55.5 ± 13.2% (*L. vaginalis* BC17, *p* < 0.0001, *n* = 5) to 93.1 ± 5.2% (*L. crispatus* BC5, *p* = 0.0001, *n* = 5) in tonsillar tissue (**Figure 2D**).

### Virucidal Capacity of *Lactobacillus*-CM against HIV-1

**FIGURE 3:**
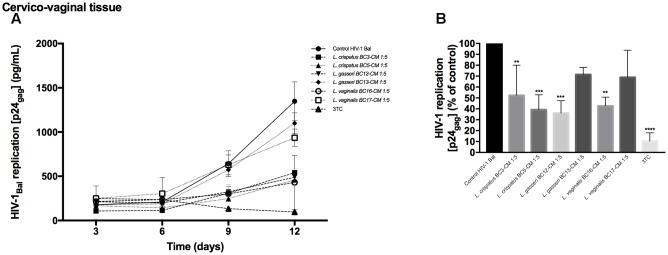
**Virucidal effect of *Lactobacillus*-CM against HIV-1.** Virucidal capacities of six strains, *L. crispatus* (BC3 and BC5); *L. gasseri* (BC12 and BC13); and *L. vaginalis* (BC16, BC17) are presented. HIV-1 preparation was pretreated with *Lactobacillus*-CM diluted 1:5 for 1 h, and HIV-1 infectivity was tested in cervico-vaginal tissues *ex vivo*. **(A)** We evaluated the kinetics of HIV-1 replication by measuring the levels of p24_gag_ in tissue culture medium. **(B)** Replication of HIV-1 in *Lactobacillus*-treated tissues expressed as percentages of HIV-1 replication in untreated control (black bars). Statistical significance vs. control is presented. Bars represent mean ± SD from tissues of five patients. Asterisks indicate statistical significance by one-way ANOVA multiple comparison (^∗^*p* < 0.05, ^∗∗^*p* < 0.01, ^∗∗∗^*p* < 0.001, ^∗∗∗∗^*p* < 0.0001). 3TC (dideoxythiacytidine or lamivudine) at 10 μM is a powerful HIV-1 inhibitor that we used in our study as a positive control.

In order to understand whether *Lactobacillus*-CM can suppress HIV-1 infectivity before interaction with tissues, we pre-incubated HIV-1 for 1 h with *Lactobacillus*-CM (diluted 1:5) and tested HIV-1 infectivity in cervico-vaginal tissues *ex vivo*. We studied the virucidal capacities of *Lactobacillus*-CM of six strains: *L. crispatus* (BC3, BC5), *L. gasseri* (BC12, BC13), and *L. vaginalis* (BC16, BC17). As shown in **Figure 3A**, HIV-1 replication was reduced when cervico-vaginal tissues were infected with HIV-1 pre-treated with *Lactobacillus*-CM from *L. crispatus* BC3 (47.7 ± 7.0%, *p* = 0.005, *n* = 5), *L. crispatus* BC5 (60.9 ± 13.8%, *p* = 0.0005, *n* = 5), *L. gasseri* BC12 (64.0 ± 11.4%, *p* < 0.0001, *n* = 5), and *L. vaginalis* BC16 (57.4 ± 8.1%, *p* = 0.003, *n* = 5) (**Figure 3B**). No statistically significant inhibition was observed due to *Lactobacillus*-CM from *L. gasseri* BC13 (28.6 ± 6.6%, *p* = 0.13, *n* = 5) and *L. vaginalis* BC17 (31.1 ± 24.8%, *p* = 0.06, *n* = 5).

### Effects of Lactic Acid and pH on HIV-1 Replication

**FIGURE 4:**
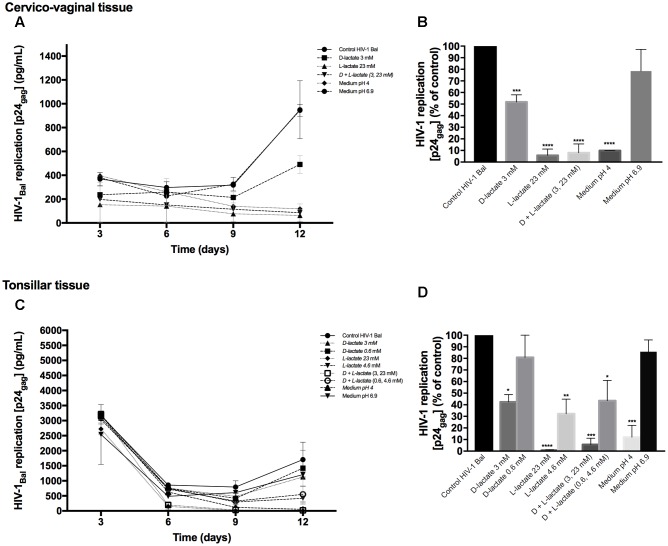
**Effect of lactic acid isomers D and L and of pH on HIV-1 replication.** The effects of lactate isomers D and L at concentrations found in *Lactobacillus*-CM on HIV-1 replication were tested in cervico-vaginal **(A,B)** and tonsillar **(C,D)** tissues. Isomers D and L were respectively tested at 3 and 23 mM concentrations, which correspond to the average concentrations of lactic acid found in *Lactobacillus*-CM. The mixture of isomers L and D was tested at the concentrations of 3 and 23 mM. We evaluated the effect of acidic pH on HIV-1 infectivity in *ex vivo* tissues by buffering the culture medium at pH 4 and pH 6.9 using HCl. **(A,C)** We evaluated the kinetics of HIV-1 replication in tissues by measuring the levels of p24_gag_ in culture medium. **(B,D)** Replication of HIV-1 in *Lactobacillus*-treated tissues was expressed as percentage of HIV-1 replication in untreated control (black bars). Statistical significance vs. control is presented. Bars represent mean ± SD from tissues of three to five donors. Asterisks indicate statistical significance by one-way ANOVA multiple comparison (^∗^*p* < 0.05, ^∗∗^*p* < 0.01, ^∗∗∗^*p* < 0.001, ^∗∗∗∗^*p* < 0.0001).

To investigate whether lactic acid produced by lactobacilli is responsible for HIV-1 inhibition, we measured the concentrations of lactic acid isomers D and L in *Lactobacillus*-CM (**Table 1**). Depending on the strain, the concentrations of lactic acid isomers D and L ranged from 1.8 to 3.6 mM and from 9.0 to 24.7 mM, respectively. *L. gasseri* BC12 was the strain that produced the highest concentrations of both isomers, while *L. crispatus* BC17 was the strain that produced the lowest concentrations. Next, we tested the effects of lactic acid isomers at the concentration found in undiluted *Lactobacillus*-CM or CM diluted 1:5 on HIV-1 replication in tissues *ex vivo* (**Figure 4**). As shown in **Figures 4A,C**, lactic acid isomers D (3 mM), L (23 mM), and D + L (3; 23 mM) significantly reduced HIV-1 replication in both cervico-vaginal and tonsillar tissues. We found that D lactate (3 mM) inhibited HIV-1 replication by 48.2 ± 6.2% in cervico-vaginal (*p* = 0.0004, *n* = 3) and by 57.6 ± 33.2% in tonsillar (*p* = 0.0125, *n* = 3) tissue cultures, while L lactate (23 mM) suppressed HIV-1 replication by 94.3 ± 5.5% (*p* < 0.0001, *n* = 3) and by 99.3 ± 21.9% (*p* < 0.0001, *n* = 3) in cervico-vaginal and tonsillar tissues, respectively. The mixture of D + L lactate suppressed HIV-1 replication by 92.1 ± 7.7% in cervico-vaginal and by 94.4 ± 30.4% in tonsillar tissue (*p* < 0.0001, *n* = 3) (**Figures 4B,D**). Afterward, we evaluated the effect of lactic acid isomers at the concentrations found in fivefold-diluted *Lactobacillus*-CM on HIV-1 replication in lymphoid tissue (**Figures 4C,D**). We found that isomer D did not inhibit HIV-1 replication while isomer L and the mixture of isomers D + L significantly reduced HIV-1 replication in tonsillar tissues by 67.8 ± 0.8% (isomer L, *p* = 0.0033, *n* = 3) and by 56.5 ± 9.5% (isomers D + L, *p* = 0.0142, *n* = 3) (**Figure 4D**).

**Table 1:** Lactic acid isomers D and L and pH in *Lactobacillus*-CM.

*Lactobacillus* strains	D-Lactate		L-Lactate		pH	
	mM	mM/10^8^ bacteria	mM	mM/10^8^ bacteria	*Lactobacillus*-CM	*Lactobacillus*-CM 1:5
*L. crispatus* BC3	3.1	0.4	15.1	2.2	4.0	6.6
*L. crispatus* BC5	3.1	0.3	24.0	2.4	3.8	6.4
*L. gasseri* BC12	3.6	0.4	24.7	2.8	3.8	6.3
*L. gasseri* BC13	3.2	0.3	22.2	2.0	3.8	6.4
*L. vaginalis* BC16	2.7	0.3	22.4	2.2	3.8	6.4
*L. vaginalis* BC17	1.8	0.4	9.0	1.9	4.6	6.9

Furthermore, we evaluated whether the effect of lactobacilli on HIV-1 replication is due to the acidic pH of the *Lactobacillus*-CM. As shown in **Table 1**, pH values of undiluted *Lactobacillus*-CM ranged from 3.8 to 4.6 and of fivefold-diluted *Lactobacillus*-CM from 6.3 to 6.9. To mimic the effect of pH on HIV-1 replication, we acidified the culture medium of human cervico-vaginal and tonsillar tissues with HCl. In the culture medium buffered to pH 4, HIV-1 replication was reduced in both cervico-vaginal (90.1 ± 0.1%, *p* < 0.0001, *n* = 3) and tonsillar tissue (88.0 ± 17.5%, *p* = 0.0003, *n* = 3) compared with control tissue blocks cultured in regular medium (**Figures 4B,D**). No statistically significant inhibition of HIV-1 replication in cervico-vaginal or tonsillar tissues was observed when the culture medium was buffered to pH 6.9 (21.6 ± 18.8%, *p* = 0.1492, *n* = 2 and 14.28 ± 10.19%, *p* = 0.9, *n* = 3, respectively).

### Virucidal Capacity of *Lactobacillus* Cells against HIV-1

**FIGURE 5:**
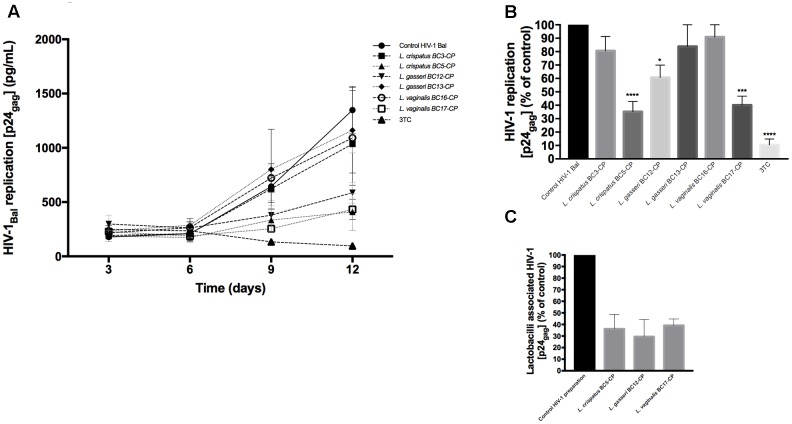
**Virucidal effect of *Lactobacillus* against HIV-1.** Virucidal capacity of six strains, *L. crispatus* (BC3, BC5), *L. gasseri* (BC12, BC13), and *L. vaginalis* (BC16, BC17) is presented. HIV-1 was pre-treated with *Lactobacillus*-CP at 10^8^ CFU/mL, and HIV-1 infectivity was then tested in cervico-vaginal tissues *ex vivo*. **(A)** We evaluated HIV-1 replication kinetics in tissues by measuring p24_gag_ in culture medium. **(B)** Replication of HIV-1 in *Lactobacillus*-treated tissues was expressed as percentage of HIV-1 replication in untreated control (black bars). **(C)** Fractions of p24_gag_ associated with CP after incubation with HIV-1 are presented. Statistical significance vs. control was calculated. Bars represent mean ± SD from five patients. Asterisks indicate statistical significance by one-way ANOVA multiple comparison (^∗^*p* < 0.05, ^∗∗^*p* < 0.01, ^∗∗∗^*p* < 0.001, ^∗∗∗∗^*p* < 0.0001). 3TC (dideoxythiacytidine or lamivudine) at 10 μM is a powerful HIV-1 inhibitor that we used in our study as a positive control.

In order to understand if vaginal *Lactobacillus* themselves are able to suppress HIV-1 infectivity, the virucidal capacities of six strains, *L. crispatus* (BC3, BC5), *L. gasseri* (BC12, BC13), and *L. vaginalis* (BC16, BC17), were studied. HIV-1 was first incubated with *Lactobacillus*-CP, and after bacteria have been washed off, the infectivity of HIV-1 was tested in cervico-vaginal tissues *ex vivo*, as described in Section “Materials and Methods.” As shown in **Figure 5A**, tissue infection with HIV-1 pre-incubated with *Lactobacillus*-CP for 1 h resulted in inhibition of HIV-1 replication by 64.7 ± 14.9% for *L. crispatus* BC5 (*p* < 0.0001, *n* = 5), by 39.3 ± 18.4% for *L. gasseri* BC12 (*p* = 0.0124, *n* = 5), and by 59.8 ± 13.2% for *L. vaginalis* BC17 (*p* = 0.0002, *n* = 5). No statistically significant inhibition was observed with *L. crispatus* BC3 (19.4 ± 18.5%, *p* = 0.46, *n* = 5), *L. gasseri* BC13 (16.1 ± 27.9%, *p* = 0.64, *n* = 5), and *L. vaginalis* BC16 (9.0 ± 15.6%, *p* = 0.96, *n* = 5) (**Figure 5B**).

Thereafter, to investigate whether this suppression of HIV-1 replication of cervico-vaginal tissue by *L. crispatus* BC5, *L. gasseri* BC12, and *L. vaginalis* BC17 in the above-described experiments was due to viral binding to *Lactobacillus* cells, we measured the concentration of p24_gag_ on *Lactobacillus*-CP after bacteria were separated by centrifugation. We found that these three strains adsorbed 36.2 ± 21.5, 29.6 ± 25.6, and 39.2 ± 9.6% of HIV-1, respectively, as evaluated from measurements of p24_gag_ (**Figure 5C**). In the CP of the remaining strains (*L. crispatus* BC3, *L. gasseri* BC13, and *L. vaginalis* BC16) the p24_gag_ was less than 10% of the original HIV-1 preparation (data not shown).

## Discussion

The human female genital tract is normally colonized by a vast number of microorganisms collectively referred to as the vaginal microbiota (Petrova et al., 2015). Although minor variations depending on age, menstruation, sexual activity, use of medication, hygiene practice, etc. (Srinivasan and Fredricks, 2008) may affect the vaginal microbiota, healthy women of reproductive age are generally dominated by *Lactobacillus* species (Pavlova et al., 2002; Hyman et al., 2005; Ravel et al., 2011). A *Lactobacillus*-dominated microbiota appears to be a biomarker for a healthy vaginal ecosystem, as changes in the vaginal microbiota, especially shifting away from *Lactobacillus* dominance, are associated with bacterial vaginosis and increased risks of acquisition of sexually transmitted infections (Cherpes et al., 2003; Wiesenfeld et al., 2003), in particular HIV (Taha et al., 1998; Atashili et al., 2008; Nardis et al., 2013; Mirmonsef and Spear, 2014; Petrova et al., 2015; Gosmann et al., 2017). Several mechanisms have been suggested to explain the protective role of *Lactobacillus* against HIV as well as the increased risk of HIV acquisition in the background of bacterial vaginosis, but all these mechanisms remain to be substantiated (Petrova et al., 2013). The vaginal microbiota seems to protect against HIV directly, by production of antiviral compounds (lactic acid, hydrogen peroxide, bacteriocins, and lectin molecules), or indirectly, stimulating immune responses or inhibiting colonization of microorganisms that cause bacterial vaginosis (Petrova et al., 2013, 2015). *Ex vivo* models may contribute to decipherment and substantiation of these mechanisms under controlled laboratory conditions.

Here, we investigated some of these mechanisms by studying the effects of vaginal lactobacilli on HIV-1 in the context of human cervico-vaginal and tonsillar tissues *ex vivo* (Saba et al., 2010; Merbah et al., 2011; Introini et al., 2014). These human tissue cultures offer major advantages over single-cell cultures, as they retain general tissue cytoarchitecture and important functional aspects of cell–cell interactions (Grivel and Margolis, 2009). Therefore, they remain a model of choice to study host–pathogen interactions (reviewed in Grivel and Margolis, 2009). These *ex vivo* tissues have proved to be useful in studies of the effect of HIV-1 copathogens on HIV-1 replication (Grivel et al., 2001; Lisco et al., 2007; Vanpouille et al., 2007) as well as in pre-clinical drug testing (Andrei et al., 2011; Vanpouille et al., 2012). Cervico-vaginal tissue *ex vivo* is a more adequate system than tonsillar tissues to study *Lactobacillus*–HIV-1 interactions, since *in vivo* human tonsillar tissues do not come into contact with lactobacilli. Nevertheless, we used not only cervico-vaginal but also tonsillar tissues, because the latter are typical lymphoid tissue, where critical events in HIV transmission and infection occur *in vivo*. Also, tonsils are more accessible and unlike cervical tissues are supplied in amounts needed to compare the effects of multiple strains of lactobacilli on HIV-1 in the same donor tissue. It is important that our results were similar for both cervico-vaginal and tonsillar tissues.

Earlier, an *ex vivo* model of porcine vaginal mucosa was used to investigate the mechanistic role of *Lactobacillus* species in colonization by *Gardnerella vaginalis* and *N. gonorrhoeae* (Breshears et al., 2015). Although the porcine vaginal mucosa system offered advantages over single-cell cultures, it may not reflect important features of the human system, in particular those of human mucosal epithelia, which are crucial for interactions of HIV with other pathogens.

To address the effects of lactobacilli on HIV-1 infection in the context of human tissues, we first colonized them *ex vivo* with 15 different strains of *Lactobacillus* that were isolated from vaginal swabs of healthy premenopausal women. These strains have been characterized for their activity against *Candida*, *C. trachomatis*, and urogenital/intestinal bacteria (Parolin et al., 2015; Nardini et al., 2016; Siroli et al., 2017).

In the present work, we found that all lactobacilli colonized and grew in human tissues *ex vivo* to densities comparable with those observed in vaginal specimens (Antonio et al., 2009; Aleshkin et al., 2011). Tissue colonization with some of the tested bacterial strains resulted in the depletion of T cells. Although this phenomenon may be relevant to the protection against HIV-1 *in vivo*, we focused our study on six strains of *Lactobacillus* (*L. crispatus* BC3, BC5; *L. gasseri* BC12, BC13; and *L. vaginalis* BC16, BC17) that did not deplete cells in tissue. We found that all these lactobacilli efficiently suppressed HIV-1 replication in human tissues *ex vivo*, and we investigated the mechanisms of this phenomenon.

First, we investigated whether lactobacilli release suppressive factors that inhibit HIV-1 replication in human tissues *ex vivo*. We found that they do indeed release factors that suppress HIV-1 replication, since the CM inhibited HIV-1 replication in human cervico-vaginal and tonsillar tissues.

Although such a medium may contain multiple inhibitory factors, we first focused on two of them; pH and lactic acid, whose roles in suppressing HIV infection were suggested earlier (Martin et al., 1985; Ongradi et al., 1990; O’Connor et al., 1995; Aldunate et al., 2013). Depending on the bacterial strain, the pH of *Lactobacillus*-CM varied from 3.8 to 4.6. We adjusted the pH of the tissue culture to these pH values, and in agreement with earlier studies (Martin et al., 1985; Ongradi et al., 1990; Ravel et al., 2011) we found that this acidification may be directly responsible for HIV-1 inhibition. Low pH (<4.5) is typical for the vaginal ecosystems *in vivo* that are dominated by *Lactobacillus* species in healthy women (Fox et al., 1973; Boskey et al., 1999; O’Hanlon et al., 2013). However, during vaginal intercourse, vaginal fluid is diluted by HIV-containing semen, resulting in neutral pH (Tevi-Benissan et al., 1997). Also, in the presence of vaginal dysbiosis (i.e., bacterial vaginosis), vaginal pH increases (Onderdonk et al., 2016). Therefore, in our experiments we diluted CM with normal media, resulting in a pH between 6.3 and 6.9; this diluted CM was still inhibitory for HIV-1 replication in human tissue *ex vivo.* Control experiments with pH 6.9 demonstrated no HIV-1 suppression, suggesting that other factors beyond lowered pH may also be important for HIV-1 inhibition, at least for some of the lactobacilli.

One such factor considered in the literature is the major *Lactobacillus* metabolite lactic acid (O’Hanlon et al., 2011, 2013). The importance of this metabolite is evidenced by the fact that in our experiments we observed a correlation between the capacity of supernatant of lactobacilli to inhibit HIV-1 replication and the capacity of lactobacilli to produce lactic acid. Therefore, we investigated the effect of lactic acid isomers D and L on HIV-1 infection. We found that the addition of these isomers to tissue culture medium at concentrations that corresponded to their amounts released by lactobacilli resulted in HIV-1 inhibition. In our work, the racemic lactic acid in *Lactobacillus*-CM ranged from 10.8 to 28.3 mM and thus was not higher than the physiological level, reported to be around 110 mM (O’Hanlon et al., 2013). The protective effect of lactic acid in our *ex vivo* tissue system is in agreement with the work of Nunn et al. (2015), who reported that a high concentration of lactic acid in cervico-vaginal mucus plays an important role in protection against HIV-1 and other sexually transmitted infections. We found that the L isomer rather than the D isomer was predominantly responsible for HIV-1 inhibition. These results indicated that lactic acid, in particular its L isomer, inhibited HIV-1 replication, independently from lowering the pH. Similarly, antibacterial properties of lactic acid against *Escherichia coli*, demonstrated earlier, were also ascribed predominantly to the L isomer (McWilliam Leitch and Stewart, 2002).

Next, we investigated whether *Lactobacillus* could have a direct virucidal effect on HIV-1. To answer this question, we incubated an HIV-1 preparation in *Lactobacillus*-CM and then tested HIV-1 infectivity in human tissue culture. We found that HIV-1 infectivity in cervico-vaginal tissue was significantly reduced. We previously reported similar findings when testing the effect of *Lactobacillus*-CM on *C. trachomatis* (Nardini et al., 2016). Finally, we investigated whether direct interactions with lactobacilli may affect HIV-1. We found that a significant fraction of virions is adsorbed on bacteria. These virucidal effects of lactobacilli may be relevant to the inhibition of HIV-1 transmission *in vivo*.

In general, the level of HIV-1 suppression may depend on the superimposition of multiple mechanisms, different for each *Lactobacillus* strain. These mechanisms include change of pH, production of lactic acid, HIV adsorption on the surface of lactobacilli, etc. However, lactic acid produced by lactobacilli in the context of human tissues *ex vivo* seems to be a major cause of HIV-1 inhibition.

Several molecular mechanisms by which this metabolite may affect HIV-1 have been suggested. It was reported that lactic acid could disrupt cellular membranes (Alakomi et al., 2000), acidify cytosol (Russell and Diez-Gonzalez, 1998), unfold proteins (Tang et al., 2003), and inhibit enzymatic activity (McWilliam Leitch and Stewart, 2002). Any of these reported effects of lactic acid might be sufficient to suppress HIV infection, e.g., by destroying the viral envelope, unfolding gp120, and/or inhibiting HIV enzymes involved in the HIV cycle (Aldunate et al., 2013).

Like any other models, our model of human tissue has some limitations, e.g., lack of tissue polarization and limited tissue survival (∼3 weeks). In the context of this study, a significant limitation was that we were not able to maintain in tissue cultures both lactobacilli and HIV-1 simultaneously, since HIV-1 requires aerobic while lactobacilli require anaerobic conditions. Therefore, in tissue culture experiments, we investigated the effect of *Lactobacillus*-CM on HIV-1.

Extrapolated to *in vivo*, our results may explain why the presence of normal vaginal microbiota, which include multiple species of *Lactobacillus*, is associated with a decreased risk of HIV acquisition in uninfected women (Atashili et al., 2008) and with lower HIV genital shedding in infected women (Sha et al., 2005; Spear et al., 2008; Mitchell et al., 2013).

The positive effects of lactobacilli on the health of the female genital tract are generating increasing interest in their use in probiotic formulations for the prophylaxis and therapy of several vaginal disturbances (Reid et al., 2001; Burton et al., 2003; Donders et al., 2010). Also, live recombinant lactobacilli releasing anti-HIV compounds have been suggested as a new therapeutic approach and successfully tested in macaques (Lagenaur et al., 2011; Brichacek et al., 2013). Further studies are needed to evaluate the potential of altering the spectra of vaginal microbiota and/or the concentrations of vaginal components such as lactic acid (Decena et al., 2006) as effective strategies to enhance vaginal health. Human tissues *ex vivo* may serve as a test system for these strategies.

## Author Contributions

RN, SZ, CV, BV, and LM designed and performed the experiments, analyzed the data, and wrote the manuscript. All the authors contributed to data interpretation. All the authors read, reviewed, and approved the final manuscript.

## Conflict of Interest Statement

The authors declare that the research was conducted in the absence of any commercial or financial relationships that could be construed as a potential conflict of interest.
